# Supplementary material for: Empirical methods for controlling false positives and estimating confidence in ChIP-Seq peaks
Source: BMC Bioinformatics. 2008 Dec 5;9:523. doi: 10.1186/1471-2105-9-523 (PMC2628906; doi:10.1186/1471-2105-9-523)
Supplement: Additional File 1 — A variety of html documents from the USeq web site detailing the available applications, their best usage, output file type descriptions, command line menus, etc. [file 1471-2105-9-523-S1.zip › USeqUserGuides/JavaDoc.zip!/JavaDoc/edu/utah/seq/parsers/BarParser.html]

BarParser


|  |  |  |
| --- | --- | --- |
| **Overview** \| **Package** \| **Class** \| **Use** \| **Tree** \| **Deprecated** \| **Index** \| **Help** | |  |
| PREV CLASS   **NEXT CLASS** | **FRAMES**    **NO FRAMES**     **All Classes** |  |
| SUMMARY: NESTED \| FIELD \| CONSTR \| METHOD | DETAIL: FIELD \| CONSTR \| METHOD |  |


---


## edu.utah.seq.parsers Class BarParser

```
java.lang.Object
  edu.utah.seq.parsers.BarParser
```

---

``` public class BarParser extends java.lang.Object ```

Class for reading and writing xxx.bar files.

**Author:**
:   Nix

    ```
       Bar format definition:

       MAIN HEADER FOR ENTIRE FILE

       1    Char    8       The file type identifier. This is always set to "barr\r\n\032\n".
       2    Float   4       The file version number.  Valid versions are 1.0 and 2.0
       3    Integer 4       The number of sequences stored in the file. Referred to as NSEQ.
       4    Integer 4       The number of columns per data point. Referred to as NCOL.
       5    Integer 4*NCOL  The field types, one per column of data. The possible values are:
                 0 - Double
                 1 - Float
                 2 - 4 byte signed integer
                 3 - 2 byte signed integer
                             4 - 1 byte signed integer
                             5 - 4 byte unsigned integer
                             6 - 2 byte unsigned integer
                             7 - 1 byte unsigned integer
       6    Integer 4       Numbern of tag/value pairs.
       7    Integer 4       The number of characters in the name of the tag. Referred to as TAGNAMELEN.
       8    Char    TAGNAMELEN      The name of the tag.
       9    Integer 4       The number of characters in the value part of the tag/value pair. Referred to as TAGVALLEN.
       10   Char    TAGVALLEN       The value of the tag/value pair.


       SECTION HEADER FOR EACH BAR SEQ/DATA 

       11   Integer 4       The number of characters in the name of the sequence. Referred to as SEQNAMELEN.
       12   Char    SEQNAMELEN      The sequence name (ie chr21).
       13   Integer 4       The number of characters in the name of the sequence group.  Referred to as SEQGROUPNAMELEN.  Used only in version 2.0 or greater.
       14   Char    SEQGROUPNAMELEN The name of the group of which the sequence is a member (for example, often specifies organism, ie Homo sapiens).  Referred to as SEQGROUPNAME.  Used only in version 2.0 or greater.
       15   Integer 4       The number of characters in the sequence version string. Referred to as SEQVERLEN.
       16   Char    SEQVERLEN       The sequence version. (ie hg18)
       17   Integer 4       Number of tag/value pairs.  Used only in version 2.0 or greater.
       18   Integer 4       The number of characters in the name of the tag. Referred to as TAGNAMELEN.  Used only in version 2.0 or greater.
       19   Char    TAGNAMELEN      The name of the tag.  Used only in version 2.0 or greater.
       20   Integer 4       The number of characters in the value part of the tag/value pair. Referred to as TAGVALLEN.  Used only in version 2.0 or greater.
       21   Char    TAGVALLEN       The value of the tag/value pair.  Used only in version 2.0 or greater.
       22   Integer 4       The number of data points defined in the sequence. Each data point will contain NCOL column values.
       23                   The next set of values in the file is the data points for the sequence. Each data point contains NCOL column values. The type, thus the size, of each column is defined above in the field types section.
    ```

    Note, several reserve xxx\_TAG names are used to put info into the tagValue pair HashMap, see below, these include:
    Strand information can be recorded '+, -, or .'
    Read length, for signature sequencers such as SOLiD and Solexa.
    Loads xxx.bar.zip compressed files too!

---

| **Field Summary** | |
| --- | --- |
| `static java.lang.String` | `BP_3_PRIME_SHIFT` |
| `static java.lang.String` | `DESCRIPTION_TAG` |
| `static java.lang.String` | `GRAPH_MAX_Y_TAG` |
| `static java.lang.String` | `GRAPH_MIN_Y_TAG` |
| `static java.lang.String` | `GRAPH_TYPE_BAR` |
| `static java.lang.String` | `GRAPH_TYPE_COLOR_TAG` |
| `static java.lang.String` | `GRAPH_TYPE_DOT` |
| `static java.lang.String` | `GRAPH_TYPE_HEATMAP` |
| `static java.lang.String` | `GRAPH_TYPE_LINE` |
| `static java.lang.String` | `GRAPH_TYPE_MINMAXAVE` |
| `static java.lang.String` | `GRAPH_TYPE_STAIRSTEP` |
| `static java.lang.String` | `GRAPH_TYPE_TAG` |
| `static java.lang.String` | `READ_LENGTH_TAG` |
| `static java.lang.String` | `SOURCE_TAG` |
| `static java.lang.String` | `STRAND_TAG`             Reserved tag names for writing to bar file tagValue pairs. |
| `static java.lang.String` | `UNIT_TAG` |
| `static java.lang.String` | `WINDOW_SIZE` |


**Constructor Summary**

`BarParser()`


| **Method Summary** | |
| --- | --- |
| `java.io.File` | `getBarFile()` |
| `int[]` | `getBasePositions()` |
| `java.lang.String` | `getChromosome()` |
| `int` | `getNumberPositionValues()` |
| `java.lang.String` | `getStrand()` |
| `java.util.HashMap<java.lang.String,java.lang.String>` | `getTagValues()` |
| `float[]` | `getValues()` |
| `java.lang.String` | `getVersionedGenome()` |
| `boolean` | `isZipCompress()` |
| `void` | `loadSimpleBarFile(boolean loadPositionValues)`             This is a complete hack of Gregg Helt's IGB code to parse bar files. |
| `boolean` | `readBarFile(java.io.File barFile, boolean loadPositionValues)`             Reads a bar file and sets the appropriate fields in the BarParser. |
| `void` | `readTagValPairs(java.io.DataInput dis, int pair_count)`             Method for parsing tag values from a binary stream. |
| `void` | `setBarFile(java.io.File barFile)` |
| `void` | `setBasePositions(int[] basePositions)` |
| `void` | `setChromosome(java.lang.String chromosome)` |
| `void` | `setStrand(java.lang.String strand)` |
| `void` | `setTagValues(java.util.HashMap<java.lang.String,java.lang.String> tagValue)` |
| `void` | `setValues(float[] values)` |
| `void` | `setVersionedGenome(java.lang.String versionedGenome)` |
| `void` | `setZipCompress(boolean zipCompress)` |
| `java.lang.String` | `toString()` |
| `boolean` | `writeBarFile(java.io.File barFile, java.lang.String chromosome, java.lang.String versionedGenome, char strand, int[] basePositions, float[] values, java.util.HashMap<java.lang.String,java.lang.String> tagValues)`             Writes a xxx.bar file to disk. |
| `void` | `writeSimpleBarFile()`             Writes a simple bar format graph using the BarParser field values. |
| `static void` | `writeTagValuePairs(java.io.DataOutputStream dos, java.util.HashMap<java.lang.String,java.lang.String> tagValuePairs)` |

**Methods inherited from class java.lang.Object**

`equals, getClass, hashCode, notify, notifyAll, wait, wait, wait`

**Field Detail**

### STRAND\_TAG

```
public static final java.lang.String STRAND_TAG
```

:   Reserved tag names for writing to bar file tagValue pairs.
    Don't use these keys for other purposes when putting extra info into the tagValue HashMap.
    These have specific meaning to the IGB browser

    **See Also:**: Constant Field Values

---


### READ\_LENGTH\_TAG

```
public static final java.lang.String READ_LENGTH_TAG
```

**See Also:**: Constant Field Values

---


### SOURCE\_TAG

```
public static final java.lang.String SOURCE_TAG
```

**See Also:**: Constant Field Values

---


### GRAPH\_TYPE\_TAG

```
public static final java.lang.String GRAPH_TYPE_TAG
```

**See Also:**: Constant Field Values

---


### GRAPH\_TYPE\_BAR

```
public static final java.lang.String GRAPH_TYPE_BAR
```

**See Also:**: Constant Field Values

---


### GRAPH\_TYPE\_DOT

```
public static final java.lang.String GRAPH_TYPE_DOT
```

**See Also:**: Constant Field Values

---


### GRAPH\_TYPE\_LINE

```
public static final java.lang.String GRAPH_TYPE_LINE
```

**See Also:**: Constant Field Values

---


### GRAPH\_TYPE\_MINMAXAVE

```
public static final java.lang.String GRAPH_TYPE_MINMAXAVE
```

**See Also:**: Constant Field Values

---


### GRAPH\_TYPE\_STAIRSTEP

```
public static final java.lang.String GRAPH_TYPE_STAIRSTEP
```

**See Also:**: Constant Field Values

---


### GRAPH\_TYPE\_HEATMAP

```
public static final java.lang.String GRAPH_TYPE_HEATMAP
```

**See Also:**: Constant Field Values

---


### GRAPH\_TYPE\_COLOR\_TAG

```
public static final java.lang.String GRAPH_TYPE_COLOR_TAG
```

**See Also:**: Constant Field Values

---


### GRAPH\_MIN\_Y\_TAG

```
public static final java.lang.String GRAPH_MIN_Y_TAG
```

**See Also:**: Constant Field Values

---


### GRAPH\_MAX\_Y\_TAG

```
public static final java.lang.String GRAPH_MAX_Y_TAG
```

**See Also:**: Constant Field Values

---


### DESCRIPTION\_TAG

```
public static final java.lang.String DESCRIPTION_TAG
```

**See Also:**: Constant Field Values

---


### UNIT\_TAG

```
public static final java.lang.String UNIT_TAG
```

**See Also:**: Constant Field Values

---


### BP\_3\_PRIME\_SHIFT

```
public static final java.lang.String BP_3_PRIME_SHIFT
```

**See Also:**: Constant Field Values

---


### WINDOW\_SIZE

```
public static final java.lang.String WINDOW_SIZE
```

**See Also:**: Constant Field Values


**Constructor Detail**

### BarParser

```
public BarParser()
```


**Method Detail**

### writeBarFile

```
public boolean writeBarFile(java.io.File barFile,
                            java.lang.String chromosome,
                            java.lang.String versionedGenome,
                            char strand,
                            int[] basePositions,
                            float[] values,
                            java.util.HashMap<java.lang.String,java.lang.String> tagValues)
```

:   Writes a xxx.bar file to disk.

    :   **Parameters:**: `barFile` - - should end with '.bar'. If zip compressing, the barFile name will be appended with the '.zip' extension.: `strand` - - either +, -, or .: `tagValues` - - optional input, can be null.

---


### readBarFile

```
public boolean readBarFile(java.io.File barFile,
                           boolean loadPositionValues)
```

:   Reads a bar file and sets the appropriate fields in the BarParser.
    Set loadPositionValues = true to load the position values or false to
    just load the header info.

---


### loadSimpleBarFile

```
public void loadSimpleBarFile(boolean loadPositionValues)
                       throws java.io.IOException
```

:   This is a complete hack of Gregg Helt's IGB code to parse bar files.
    It only works on single graph type files, ie two columns, int and float,
    one sequence. Reads xxx.bar or zip compressed xxx.bar.zip files.

    :   **Parameters:**: `loadPositionValues` - - set to true if you want to load the actual positions and values, otherwise just the header is loaded. **Throws:**: `java.io.IOException`

---


### readTagValPairs

```
public void readTagValPairs(java.io.DataInput dis,
                            int pair_count)
                     throws java.io.IOException
```

:   Method for parsing tag values from a binary stream.

    :   **Throws:**: `java.io.IOException`

---


### writeSimpleBarFile

```
public void writeSimpleBarFile()
                        throws java.io.IOException
```

:   Writes a simple bar format graph using the BarParser field values.
    Based on Gregg Helt's IGB code.

    :   **Throws:**: `java.io.IOException`

---


### writeTagValuePairs

```
public static void writeTagValuePairs(java.io.DataOutputStream dos,
                                      java.util.HashMap<java.lang.String,java.lang.String> tagValuePairs)
                               throws java.io.IOException
```

:   **Throws:**: `java.io.IOException`

---


### toString

```
public java.lang.String toString()
```

:   **Overrides:**: `toString` in class `java.lang.Object`

---


### getBarFile

```
public java.io.File getBarFile()
```

---


### setBarFile

```
public void setBarFile(java.io.File barFile)
```

---


### getBasePositions

```
public int[] getBasePositions()
```

---


### setBasePositions

```
public void setBasePositions(int[] basePositions)
```

---


### getChromosome

```
public java.lang.String getChromosome()
```

---


### setChromosome

```
public void setChromosome(java.lang.String chromosome)
```

---


### getStrand

```
public java.lang.String getStrand()
```

---


### setStrand

```
public void setStrand(java.lang.String strand)
```

---


### getTagValues

```
public java.util.HashMap<java.lang.String,java.lang.String> getTagValues()
```

---


### setTagValues

```
public void setTagValues(java.util.HashMap<java.lang.String,java.lang.String> tagValue)
```

---


### getValues

```
public float[] getValues()
```

---


### setValues

```
public void setValues(float[] values)
```

---


### getVersionedGenome

```
public java.lang.String getVersionedGenome()
```

---


### setVersionedGenome

```
public void setVersionedGenome(java.lang.String versionedGenome)
```

---


### isZipCompress

```
public boolean isZipCompress()
```

---


### setZipCompress

```
public void setZipCompress(boolean zipCompress)
```

---


### getNumberPositionValues

```
public int getNumberPositionValues()
```


---


|  |  |  |
| --- | --- | --- |
| **Overview** \| **Package** \| **Class** \| **Use** \| **Tree** \| **Deprecated** \| **Index** \| **Help** | |  |
| PREV CLASS   **NEXT CLASS** | **FRAMES**    **NO FRAMES**     **All Classes** |  |
| SUMMARY: NESTED \| FIELD \| CONSTR \| METHOD | DETAIL: FIELD \| CONSTR \| METHOD |  |


---
